# Inhibition of Autophagy Maintains ESC Pluripotency and Inhibits Primordial Germ Cell Formation in Chickens

**DOI:** 10.1155/2023/4956871

**Published:** 2023-04-04

**Authors:** Ying Ding, Juanjuan Zhao, Xianshuai Xu, Qisheng Zuo, Yani Zhang, Kai Jin, Wei Han, Bichun Li

**Affiliations:** ^1^Key Laboratory of Animal Genetics, Breeding and Molecular Design of Jiangsu Province, College of Animal Science and Technology, Yangzhou University, Yangzhou 225009, China; ^2^Joint International Research Laboratory of Agriculture and Agri-Product Safety of the Ministry of Education of China, Yangzhou University, Yangzhou 225009, China; ^3^Poultry Research Institute, Chinese Academy of Agricultural Science/Jiangsu Institute of Poultry Science, Yangzhou 225009, China

## Abstract

Autophagy plays an important role in the pluripotency and differentiation of stem cells. Transcriptome data showed that the autophagy genes *MAP1LC3A* and *MAP1LC3B* were significantly upregulated in primordial germ cells (PGCs). The Kyoto Encyclopedia of Genes and Genome (KEGG) results showed that the lysosome signaling pathway, which is related to autophagy, was significantly enriched in PGCs. Quantitative RT-PCR, western blotting, and transmission electron microscopy (TEM) results showed that autophagy was expressed in both embryonic stem cells (ESCs) and PGCs but was significantly activated in PGCs. To explore the role of autophagy in the differentiation of chicken ESCs into PGCs, autophagy was activated and inhibited using rapamycin and bafilomycin A1, respectively. Results of qRT-PCR, flow cytometry, and indirect immunofluorescence showed that the efficiency of PGC formation significantly decreased after autophagy inhibition. Our results showed, for the first time, that autophagy plays an indispensable role in the formation of chicken PGCs, which lays the foundation for studying the mechanism of autophagy in chicken PGCs and in bird gene editing and the rescue of endangered birds.

## 1. Introduction

Spermatogonia and oogonia are derived from primordial germ cells (PGCs), which are responsible for transmitting genetic information between generations [1, 2]. The injection of gene-edited PGCs into recipient chickens is an effective method for producing transgenic chickens. Therefore, the study of PGCs is of great significance for the genetic engineering, somatic reprogramming, and conservation of rare poultry. However, the regulatory mechanism of the developmental process of PGCs remains unclear, the key regulatory factors affecting their formation have not yet been identified, and there is an urgent need to optimize the culture system of PGCs in vitro [3–5].

Autophagy is a conserved intracellular degradation process that is important for maintaining cellular homeostasis in simple yeasts and complex mammals [6]. Autophagy is initiated by the activation of the ULK1 complex (involving ULK1, ULK2, ATG13, and FIP200). Next, the ATG5-ATG12 complex binds to ATG16 to expand the autophagosome membrane and LC3, and members of the GABARAP family bind to lipid phosphatidylethanolamine (PE) and are recruited to the membrane. ATG4B binds to ATG7 and combines with LC3-I and PE to form LC3-II. Eventually, autophagosomes fuse with lysosomes, and their contents are degraded. Degraded products such as amino acids can be reused. The large capacity of autophagosomes enables them to wrap around and remove damaged organelles and proteins, thereby maintaining normal organelle function and protein quality.

Autophagy is necessary for stem cell maintenance in mice [7]. When *ATG7* is knocked down in hematopoietic stem cells, mitochondrial superoxide levels, DNA damage, and apoptosis increase. ATG-deficient cells cannot form subclones in vitro [8, 9]. Most of the current research on autophagy in embryonic stem cells (ESCs) has been conducted in mice. The lack of autophagy in ESCs may lead to the accumulation of damaged cell components during embryonic development, which affects cell differentiation. To some extent, the ubiquitin-proteasome system may compensate for the absence of autophagy in ESCs. Proteins damaged by carbonylation accumulate in mouse ESCs and are cleared during differentiation, which may be associated with increased proteasome activity [8]. Activated autophagy observed during differentiation may contribute to the removal of damaged proteins. Cells that secreted maintenance factors from mouse embryonic fibroblasts were cultured in an unconditioned medium for three days and spontaneously differentiated. Compared to cells cultured in a conditioned medium, GFP-LC3 fluorescence spots in cells were significantly increased [10]. The number of green, fluorescent spots indicating LC3 increased rapidly after 2 h of rapid differentiation induced by the addition of the TGF-*β*/TGF-*β* receptor II inhibitor SB431542. These findings suggest a relationship between autophagy and cell differentiation. In addition, autophagy is the key to the differentiation of mononuclear macrophages. When *BECLIN1*/*ATG5/ATG7* were absent, the differentiation of human monocyte macrophages induced by CSF1 was blocked. Moreover, when autophagy is inhibited by 3-MA or CQ, human monocytes no longer respond to CSF2 by differentiating into macrophages [11–14]. These studies suggest that autophagy plays a regulatory role in cell differentiation, particularly in mammals.

Our results indicate that autophagy exists in ESCs and PGCs and is highly expressed in PGCs that are differentiated from ESCs. In this process, the inhibition of autophagy by Baf-A1 significantly inhibits PGCs formation, suggesting that autophagy is essential for PGCs formation. To our knowledge, this is the first study to elucidate the role of autophagy in the differentiation of chicken ESCs into PGCs. Our results provide new insights into the mechanisms underlying germ cell development in poultry and may help attenuate sterility and insufficient egg production in poultry.

## 2. Materials and Methods

### 2.1. Ethics Approval

Animal experiments were approved by the Institutional Animal Care and Use Committee of the Yangzhou University Animal Experiments Ethics Committee (permit number: SYXK [Su] IACUC 2012-0029). All experimental procedures were performed in strict adherence to the Regulations of the Administration of Affairs Concerning Experimental Animals approved by the State Council of the People's Republic of China.

### 2.2. Cell Isolation and Culture

Fertilized breeder eggs were obtained from the Poultry Research Institute (Chinese Academy of Agricultural Sciences, Yangzhou, China). Blastoderm cells from the embryonic region of the X-stage fertilized eggs were used to isolate ESCs. Isolated blastoderm cells were cultured at 37°C with 5% CO_2_ for 24 h for purification. PGCs were isolated from the genital ridge of chicken embryos which are incubated for 4.5 days. The isolation and culture methods for chicken ESCs and PGCs have been described previously [15–17].

### 2.3. Bone Morphologenic Protein 4 (BMP4) Induction System In Vitro

Purified ESCs were collected, centrifuged at 1,200 rpm/min for 6 min, resuspended in ESC medium, and inoculated into 24-well plates at a density of 10^5^ cells/well. When the cells were in good condition and the cell confluence reached approximately 70%, the ESCs were cultured in BMP4 induction medium, which contained DMEM high glucose medium, 0.1 mmol/L *β*-mercaptoethanol, 40 ng/mL human recombinant BMP4, 0.4% nonessential amino acids, 0.5% penicillin, 10% FBS, and 2% chicken serum [16]. Fresh medium was added every two days.

### 2.4. RNA-Sequencing

Total RNA was extracted from ESCs and PGCs using the TRIzol reagent (Tiangen, China). RNase-free DNase I (Takara, Dalian, China) was added to the reaction mixture for 10 min to effectively remove genomic DNA, and RNA purity and concentration were determined using a NanoDrop 2000 spectrophotometer (Thermo Scientific, USA). RNA integrity was assessed using the Agilent 2100 Bioanalyzer (Agilent Technologies, Santa Clara, CA, USA). The library was constructed, and subsequent detection was performed by Shanghai OE Biotechnology (Shanghai, China). Six sequencing libraries were constructed using an Illumina platform. In total, the effective data of each sample obtained by RNA-seq were 6.57G, 6.95G, 6.62G, 6.36G, 6.48G, and 6.85G. The Q30 bases of ESCs and PGCs were 92.46% and 91.99%, respectively, and the GC content was 50.32% and 50.26%, respectively. Principal components and box diagram analyses are available online (https://cloud.oebiotech.cn/task/). PC1 accounted for 98.03% of the total data.

### 2.5. qRT-PCR

Total RNA was extracted from the cells using TRIzol reagent (Tiangen, China). A first-strand cDNA synthesis kit (Tiangen, China) was used to synthesize cDNA according to the manufacturer's instructions. Real-time PCR experiments were performed using the SYBR Green Fluorescence Quantification Kit (Tiangen, China) according to the manufacturer's instructions. Chicken *β-actin* was used as an internal control. The relative gene expression was calculated using the 2^-*ΔΔ*Ct^ method. After qRT-PCR, the dilution ratio log value of the template series was plotted as the *x*-axis, the corresponding Ct value was plotted as the *y*-axis, and a standard curve was constructed. After constructing a standard curve, the amplification efficiency of the primers was calculated as *E* = 10^−1/slop^ − 1.

### 2.6. Blunt Injection of Chicken Embryo

The fertilized eggs were allowed to stand for 6 h, after which the blunt ends were sterilized with 75% alcohol, irradiated by a flashlight to locate the air chamber, dissolved in rapamycin (1 mg/kg) and bafilomycin A1 (0.1 mg/kg) with DMSO, injected into the chicken embryos (100 *μ*L), sealed with paraffin, and the eggs were returned to the incubator. Genital ridges were isolated for further processing when the chicken embryos had developed for 4.5 days.

### 2.7. Western Blot

A radioimmunoprecipitation assay (RIPA) lysate containing protease inhibitors was added to the cells, which were then lysed for 40 min on ice. The cells were centrifuged at 12,000 × *g* for 15 min at 4°C, and the supernatant was removed. The protein concentration was determined using a bicinchoninic acid assay protein concentration assay kit. RIPA and 6 × SDS loading buffer were added, and the samples were denatured at 100°C for 10 min. The samples were electrophoresed at 80 V for 10 min, followed by 120 V for 60 min. The films were placed in the order of filter paper, gel, PVDF film, and filter paper, and transferred at 11 V voltage. Next, 5% skim milk powder was added, and the cells were placed in a shaker and incubated for 2 h. The antibody was added to the incubation box, shaken for 1 h, and incubated overnight at 4°C. The secondary antibody was added and incubated for 2 h on a shaker at room temperature. A diaminobenzidine chromogenic solution was added to the surface, and images were captured.

### 2.8. Paraffin Sections and Glycogen Staining

The hatched chick embryos were collected in centrifuge tubes after 4.5 days, fixed with a fixative, and dehydrated after 24 h of fixation. The embryos were then placed in xylene I for transparency for 15 min and xylene II for transparency for 15 min. The transparent chicken embryos were soaked in wax at 65°C for 1 h and placed in a preheated embedding frame, and hot wax was poured into the embedding frame. Preheated tweezers were used to center the chicken embryos and remove air bubbles. After the wax was completely solidified, it was removed and trimmed, and the trimmed wax block was placed on a microtome for slicing. The slices were displayed in a 40% alcohol aqueous solution, and the slices were placed in warm water at 42°C for 1-2 s before the sections were attached to the slides. The slides were placed in a slide holder at 65°C to bake overnight, and the slides were deparaffinized. After deparaffinization and rehydration, the sections were air-dried and processed using a Periodic Acid Schiff Staining Kit (Solarbio, Item No: G1281).

### 2.9. Flow Cytometry

The cells were collected and washed with phosphate-buffered saline (PBS). Next, 1% Triton-100 was added for 20 min to permeabilize the membrane, and the cells were washed with PBS and blocked with PBS containing 10% FBS for 2 h. DDX4 antibody was added and incubated at 4°C overnight, followed by incubation at 37°C for 2 h. Cells were detected using flow cytometry.

### 2.10. Transmission Electron Microscopy (TEM)

The collected cells were fixed in Karnovsky's fixative (phosphate-buffered 2% glutaraldehyde and 2.5% paraformaldehyde) and 2% osmium tetroxide. Potassium ferricyanide (3%) was then added for 30 min. After washing with water, samples were stained with uranyl acetate. The samples were dehydrated with ethanol, acetonitrile was used as the transition fluid, and the samples were embedded in epoxy using the Epon substitute Lx112. Ultrathin sections were stained with uranyl acetate and lead citrate.

### 2.11. Alkaline Phosphatase Staining

The collected cells were washed with PBS and fixed for 3 min. Alkaline phosphatase was added, the cells were washed with PBS, and incubated in the dark for 15–20 min. Nuclear fast red was added, and cells were stained for 3–5 min after washing with PBS. Finally, the specimens were observed under a microscope.

### 2.12. Statistical Analysis

All experiments were repeated three times, and the data are presented as the mean ± standard error. Differences between groups were analyzed using *t*-tests. Statistical significance was set at *p* < 0.05. Statistical analyses were performed using GraphPad Prism 7 software (https://www.graphpad.com/scientific-software/prism/).

## 3. Results

### 3.1. Separation and Identification of ESCs and PGCs

**Figure 1:**
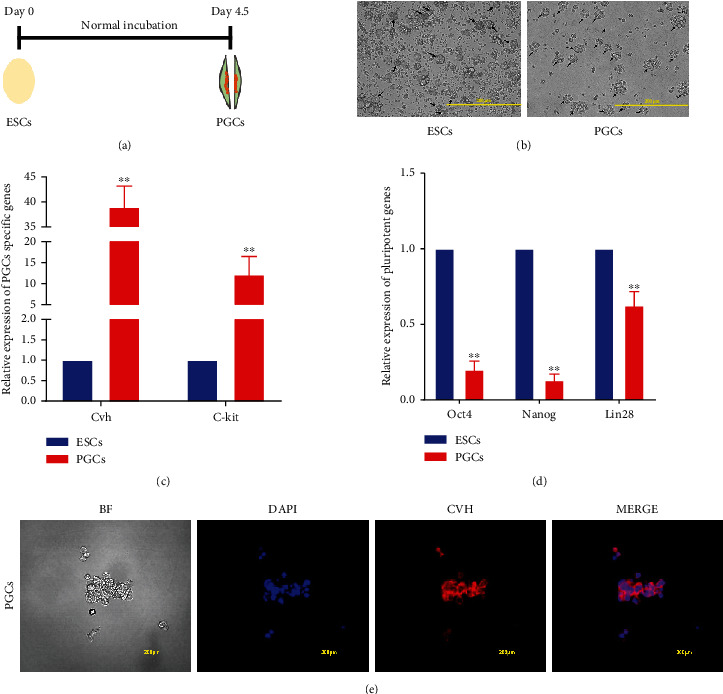
Separation and identification of ESCs and PGCs. (a) Diagram of ESCs developing into PGCs. (b) Cell morphology of chicken ESCs and PGCs. Scale bars = 200 *μ*m. (c, d) Detection of pluripotent genes and PGCs specific genes expression by qRT-PCR in ESCs and PGCs. ^∗^*p* < 0.05, ^∗∗^*p* < 0.01. (e) PGCs were stained against CVH with a rabbit monoclonal antibody.

To further explore the regulatory factors affecting PGCs formation, we conducted RNA-seq after collecting and identifying ESCs and PGCs. ESCs were collected on day 0 and PGCs from the genital ridge of chicken embryos were collected on day 4.5 (Figures 1(a) and 1(b)). We detected the expression of PGC-specific genes (*Cvh* and *C-kit*) and the pluripotency genes (*Oct4*, *Nanog*, and *Lin28*) using qRT-PCR (Table 1). The results showed that *Cvh* and *C-kit* levels were significantly higher in PGCs, whereas *Oct4*, *Nanog*, and *Lin28* levels were significantly higher in ESCs (Figures 1(c) and 1(d)). The PGC-specific protein CVH was labeled using indirect immunofluorescence, and the results showed that PGCs were successfully isolated (Figure 1(e)). Therefore, these isolated samples could be used for RNA-seq analysis.

### 3.2. Quality Assessment of RNA-Seq Data

**Figure 2:**
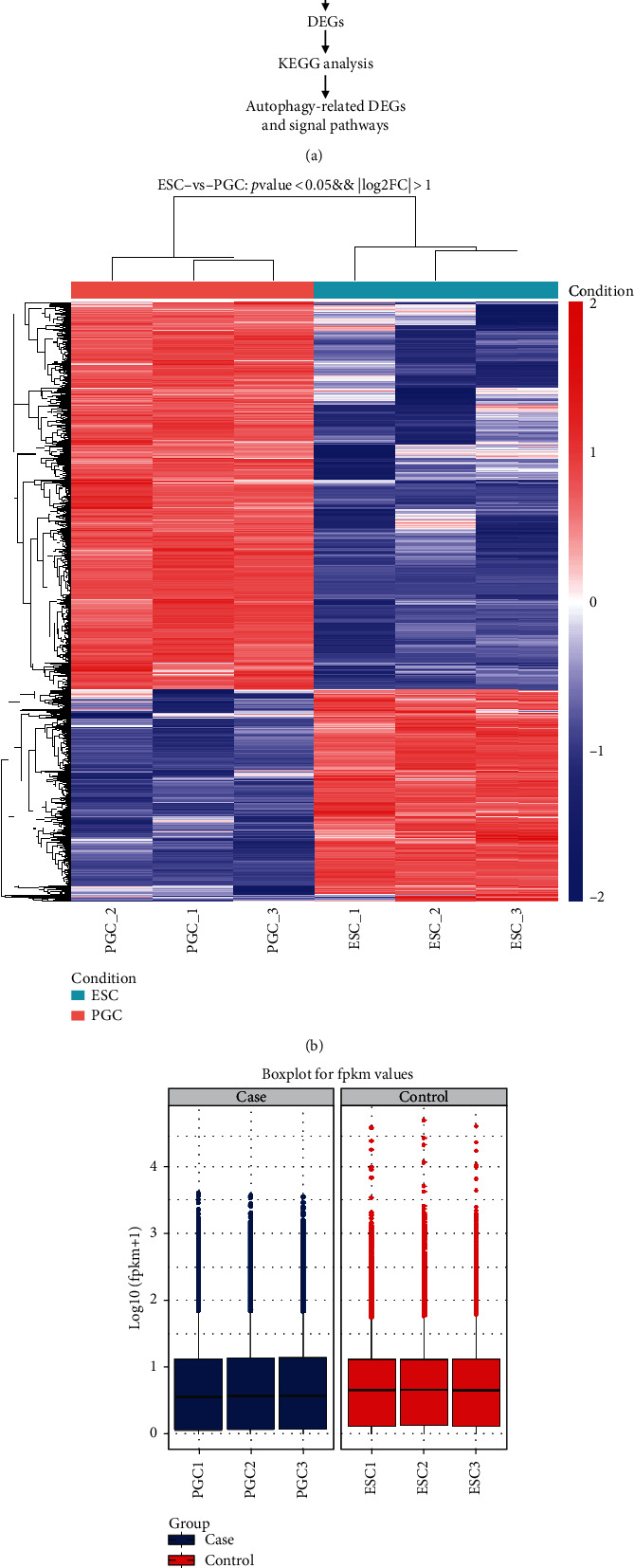
Analysis of cell transcriptome sequencing data. (a) Schematic diagram of RNA-seq analysis of ESCs and PGCs. DEGs: differentially expressed genes; KEGG: Kyoto Encyclopedia of Genes and Genomes. (b) Heat map analysis of ESCs and PGCs. (c) Box plots of ESCs and PGCs.

A heat map illustrating the hierarchical clustering of RPKM values was generated to visualize the overall gene expression patterns between ESCs and PGCs (Figure 2(b)). The box diagram shows that the expressed genes differed considerably between ESCs and PGCs (Figure 2(c)). These results showed that there were 17,444 differential genes between ESCs and PGCs, of which 5,025 were significantly different (*p* < 0.05, FC > 2).

### 3.3. Enrichment Analysis of Differentially Expressed Genes (DEGs) in ESCs and PGCs

**Figure 3:**
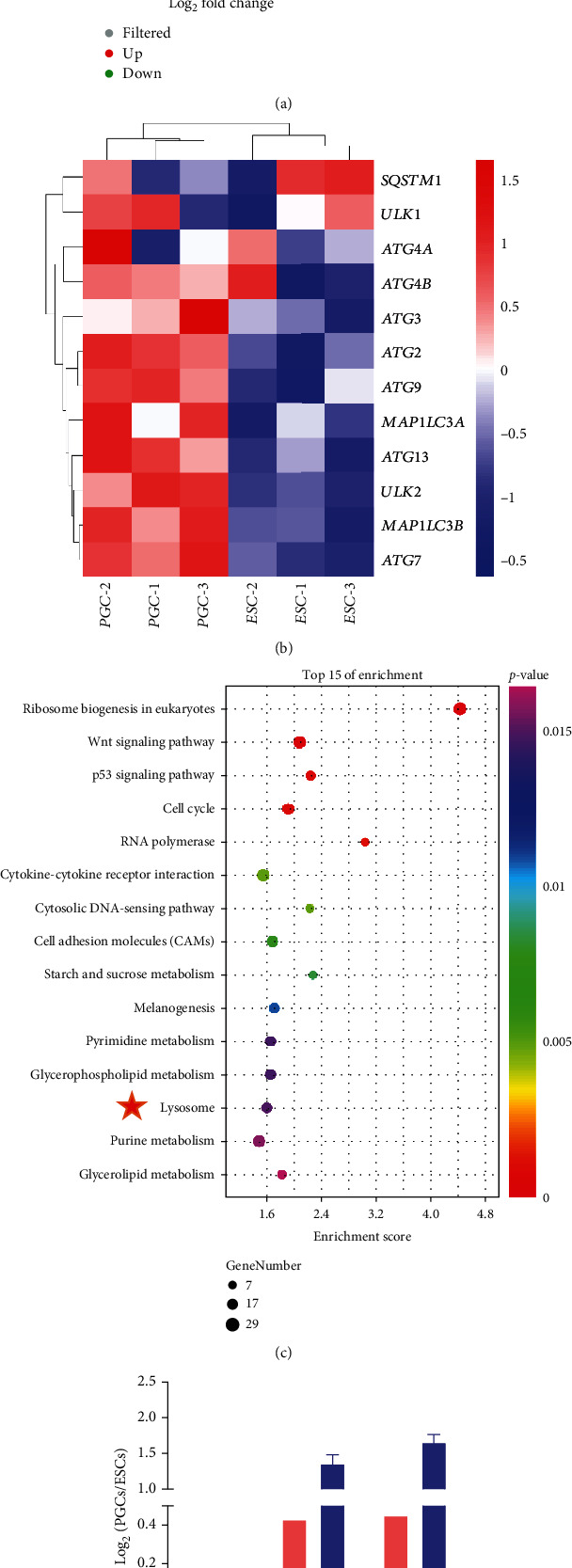
Enrichment analysis of DEGs in ESCs and PGCs in chicken. (a) Volcano map of DEGs between ESCs and PGCs. The red dots represent significantly upregulated genes in PGCs; the green dots represent significantly downregulated genes. Genes that are not differentially expressed are shown in gray. (b) Heat map showing DEGs between ESCs and PGCs. Blue represents weakly expressed genes, and red represents highly expressed genes. (c) KEGG enrichment analysis of the DEGs. (d) Expression comparisons of autophagy genes detected by RNA-seq and qRT-PCR between ESCs and PGCs.

To further explore the differences between ESCs and PGCs, we analyzed DEGs between the two groups. A total of 5,025 genes were screened and identified as significantly different (*p* < 0.05, FC > 2), accounting for 28.81% of the total genes. Volcanic map analysis showed that 3,254 genes were upregulated and 1,771 genes were downregulated in PGCs (Figure 3(a)). Further cluster analysis of these DEGs showed that the expression of autophagy genes in PGCs was higher than that in ESCs, especially *MAP1LC3A* and *MAP1LC3B* (Figure 3(b)). KEGG analysis of the DEGs between ESCs and PGCs (Figure 3(c)) showed that the lysosome-signaling pathway, which is related to autophagy, was significantly enriched. Based on the above sequencing results, we verified the expression of autophagy related genes in ESCs and PGCs by qRT-PCR, and the results were consistent (Figure 3(d)).

### 3.4. Autophagy Was Differentially Expressed in ESCs and PGCs

**Figure 4:**
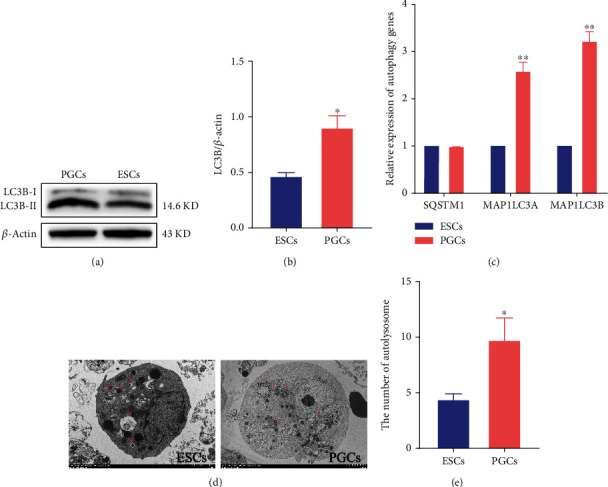
Detection of autophagy in ESCs and PGCs. (a, b) The expression of autophagy related protein in ESCs and PGCs was detected using western blot and the gray scale analysis of western blot. (c) The expression of autophagy related genes in ESCs and PGCs was detected by qRT-PCR. ^∗^*p* < 0.05, ^∗∗^*p* < 0.01 (d) Autolysosomes in ESCs and PGCs were observed under transmission electron microscopy, marked with red arrows. (e) The number of autolysosomes in ESCs and PGCs.

Based on these results, we hypothesized that autophagy is differentially expressed in ESCs and PGCs. We detected the expression of the autophagy marker protein LC3B in ESCs and PGCs by western blotting, and the results showed that LC3B was highly expressed in PGCs (Figures 4(a) and 4(b)). The qRT-PCR results showed that the autophagy related genes *MAP1LC3A* and *MAP1LC3B* were highly expressed in PGCs (Figure 4(c)). We observed the number of autolysosomes in ESCs and PGCs using transmission electron microscopy and found that there were more autolysosomes in PGCs (Figures 4(d) and 4(e)). In conclusion, autophagy was significantly activated in PGCs, further indicating that it may be involved in PGCs formation.

### 3.5. Inhibition of Autophagy Inhibits PGCs Formation in Chicken Embryos In Vivo

**Figure 5:**
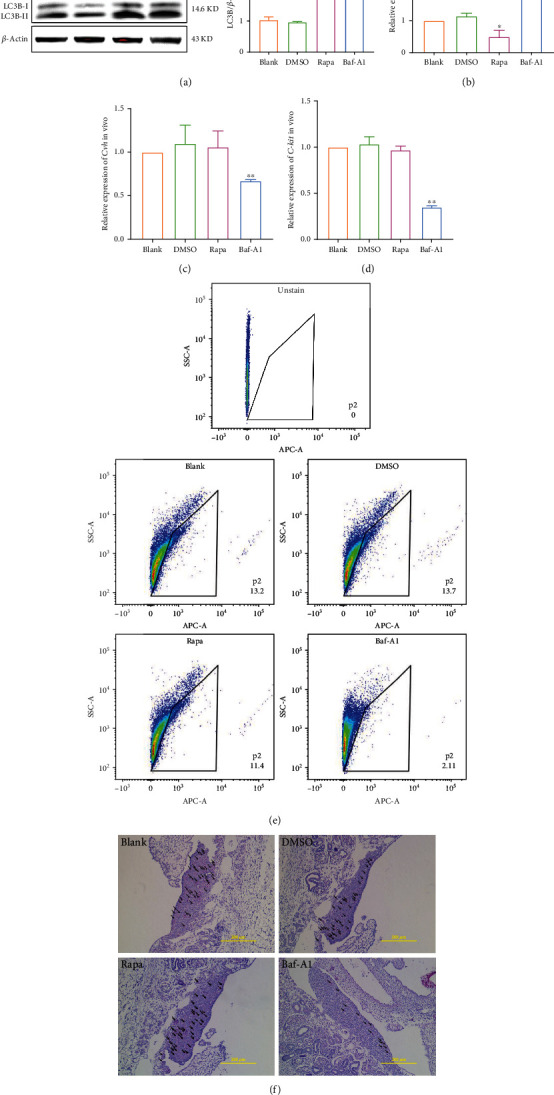
Baf-A1 inhibited the formation of PGCs in vivo. (a, b) After rapamycin (autophagy activator) or Baf-A1 (autophagy inhibitor) was injected in vivo, the expression of p62 and LC3B was detected using qRT-PCR and western blot. (c, d) The expression of reproductive marker genes was detected using qRT-PCR. (e) After labelling positive cells with CVH (PGCs specific protein), the efficiency of PGCs formation after autophagy activation or inhibition in vivo was detected by flow cytometry. (f) After rapamycin (autophagy activator) or Baf-A1 (autophagy inhibitor) was injected in vivo, the number of PGCs in chicken embryos between groups was calculated using glycogen staining of paraffin sections. Scale bar = 200 *μ*m.

Figure 4 showed that autophagy is differentially expressed in ESCs and PGCs, whereas previous studies have shown that autophagy is involved in mammalian cell differentiation [8–14], suggesting that autophagy may also play an important regulatory role in the formation of chicken PGCs. Therefore, we further explored the role of autophagy in PGCs formation. Rapamycin and Baf-A1 were injected into fertilized eggs on day 0, and the genital ridges were removed from the chicken embryos on day 4.5 to detect autophagy and the number of PGCs in vivo. By detecting LC3B and *p62*, we found that autophagy was activated by rapamycin and inhibited by Baf-A1 (Figures 5(a) and 5(b)). The qRT-PCR results showed that when autophagy was activated, *Cvh* and *C-kit* gene expression levels did not change. However, when autophagy was inhibited by Baf-A1, *Cvh* and *C-kit* gene expression were downregulated (Figures 5(c) and 5(d)). The formation of PGCs in each group was detected using flow cytometry by labelling with CVH, a specific PGC protein. The results showed that the formation of PGCs did not change when autophagy was activated compared to that in the control group; however, PGCs formation was only 2.11% when autophagy was inhibited (Figure 5(e)). The PGCs in the different groups of chicken embryos were counted after paraffin sectioning and glycogen staining. The results showed that the number of PGCs in the Baf-A1 group was reduced (Figure 5(f)).

### 3.6. Inhibition of Autophagy Inhibits PGCs Formation In Vitro

**Figure 6:**
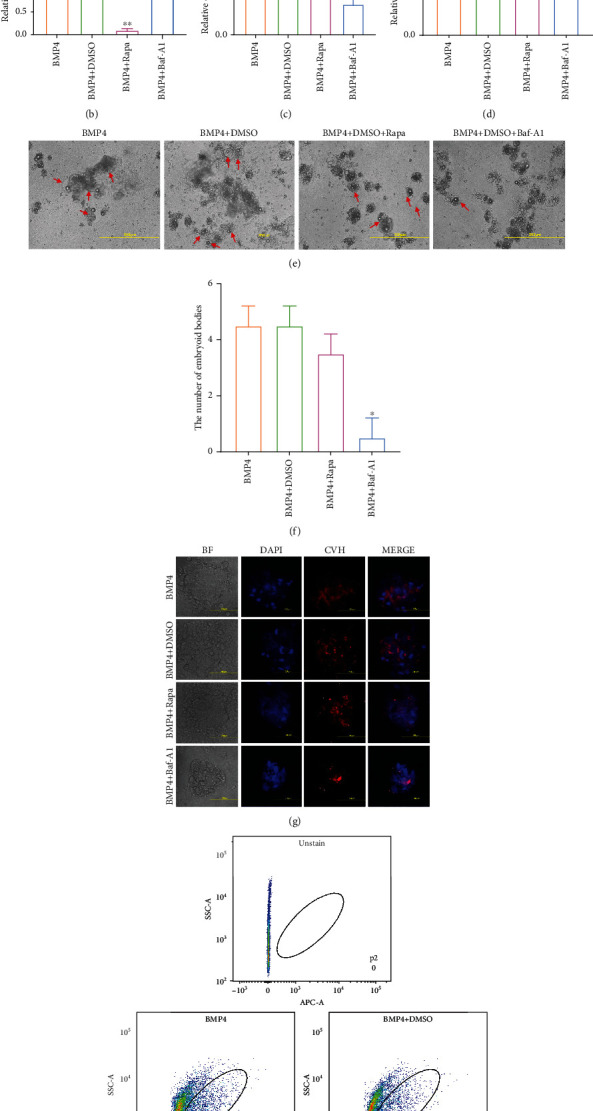
Baf-A1 inhibited the formation of PGCs in vitro. (a, b) After rapamycin (autophagy activator) or Baf-A1 (autophagy inhibitor) was added in vitro, the expression of p62 and LC3B were detected using qRT-PCR and western blot. (c, d) After the autophagy activity was altered in vitro, the expression of reproductive marker genes were detected using qRT-PCR ^∗^*p* < 0.05, ^∗∗^*p* < 0.01. (e, f) Morphological observation of the embryoid body in vitro after activation or inhibition of autophagy. Scale bar = 200 *μ*m. (g) iPGCs were stained with a rabbit monoclonal antibody against CVH. (h) Flow cytometry assays the efficiency of PGCs formation after autophagy was activated or inhibited in vitro.

Based on the BMP4 induction system previously constructed by our research group, we induced ESCs using a BMP4 induction system supplemented with rapamycin and Baf-A1. On day 4 of induction, qRT-PCR and western blotting results showed that autophagy was activated or inhibited (Figures 6(a) and 6(b)). The number of embryoid bodies was considerably reduced after autophagy inhibition (Figures 6(e) and 6(f)). Samples from each group were collected on day 6 of induction. By labelling iPGCs with CVH, indirect immunofluorescence results showed that the number of iPGCs in the BMP4+Baf-A1 group was considerably less than that in the BMP4 group (Figure 6(g)). Induction efficiency was determined using flow cytometry. The results showed that there was no change after autophagy was activated, whereas autophagy was considerably reduced after inhibition of Baf-A1 (Figure 6(h)). The qRT-PCR results were consistent (Figures 6(c) and 6(d)). These results indicated that autophagy is critical for PGCs formation, both in vivo and in vitro.

### 3.7. Inhibition of Autophagy Maintains the Pluripotency of ESCs

**Figure 7:**
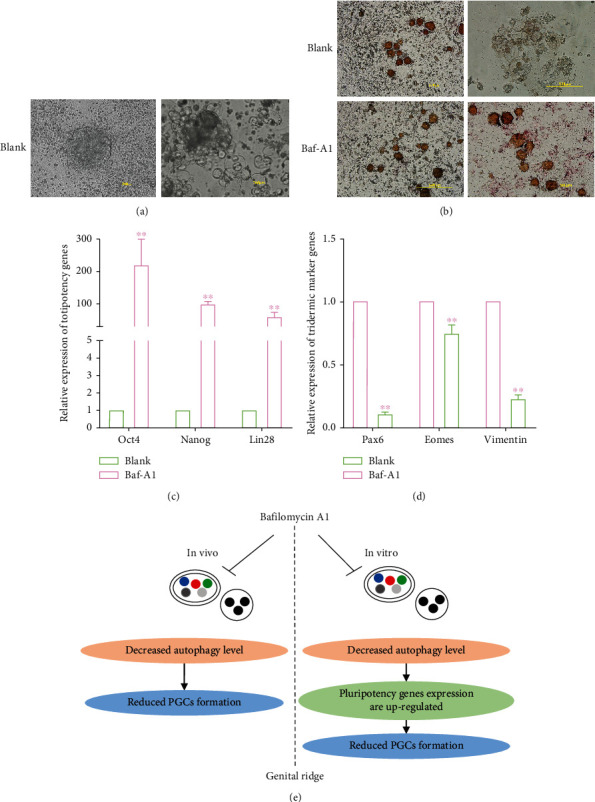
Inhibition of autophagy maintains the pluripotency of ESCs. (a) Formation of embryonic body in vitro in the absence of LIF. Scale bars = 200 *μ*m. (b) ESCs clones were stained using an alkaline phosphatase kit. Scale bars = 200 *μ*m. (c, d) The expression of pluripotency genes and tridermic marker genes in ESCs were detected by qRT-PCR. (e) A schematic diagram depicting how autophagy regulates PGCs formation in chicken.

To further explore why inhibition of autophagy inhibits the formation of PGCs, we added Baf-A1 to ESCs cultured without leukemia inhibitory factor (LIF) to explore whether autophagy affects ESCs differentiation. As shown in Figure 7(a), ESCs began to differentiate and form a large number of embryoid bodies when cultured without LIF. Interestingly, when the autophagy inhibitor Baf-A1 was added, the expression of ESCs pluripotency genes *Oct4*, *Nanog*, and *Lin28* increased considerably (Figure 7(c)). Tridermic genes were also greatly downregulated (Figure 7(d)). The alkaline phosphatase results were consistent with the qRT-PCR results (Figure 7(b)).

## 4. Discussion

Stem cell differentiation refers to morphological and functional remodeling, which is accomplished through the dynamic coordination of macromolecular degradation and synthesis, as well as transcription and epigenetic reprogramming [18–21]. Autophagy plays a key role in dynamic stem cell homeostasis. Macromolecular degradation in differentiated stem cells occurs through macroautophagy, which involves the encapsulation of cellular components such as organelles, membranes, and cytoplasmic proteins in the autophagosome, which eventually merges with the lysosome to form an autolysosome, which eventually degrades the contents. There is evidence that self-renewal, pluripotency, and differentiation of stem cells depend on autophagy [22]. Based on this, we examined autophagy in ESCs and PGCs. Both western blotting and transmission electron microscopy results showed that autophagy was present in both types of stem cells. However, there have been no reports on the involvement of autophagy in the differentiation of chicken ESCs through the coordination of macromolecular degradation and synthesis. This study provides a theoretical foundation for further exploration of the functions and specific regulatory mechanisms of autophagy in chicken germ stem cells.

Many studies have shown that autophagy is involved in mammalian cell differentiation, especially in red blood cells (RBCs). As RBCs mature, autophagy degrades organelles and proteins, leaving only hemoglobin, which allows the cells to contract and squeeze through the smallest capillaries. When the nucleus is expelled from differentiated RBCs, mitochondrial clearance occurs via autophagy. Loss of *BNIP3L*, *ULK1*, or *ATG7* leads to mitosis disorder in RBCs and ultimately leads to severe developmental and functional deficits, including those related to mitochondrial retention [23, 24]. These results suggest that autophagy promotes cell differentiation and requires large-scale remodeling to adapt to specific cellular functions. In addition, scientists have explored changes in autophagic activity during cell differentiation and the effect of autophagy on cell differentiation. Zeng et al. [25] found that autophagy was highly activated in hepatic progenitor cells and gradually decreased during the progression of hepatic differentiation. Li et al. [26] confirmed that VK2 activates autophagy in MC3T3 cells and promotes their differentiation. Vidoni et al. [18] studied the role of autophagy in the osteogenic differentiation of HGMSCs. The results showed that resveratrol alone did not induce osteoblastic differentiation but significantly induced autophagy. Resveratrol accelerates the differentiation of human gingival mesenchymal stem cells into osteoblasts by activating autophagy in combination with osteogenic differentiation-inducing factors. Interestingly, in this study, LC3B, a key protein involved in autophagy, was differentially expressed in ESCs and PGCs. This finding was consistent with our transcriptome data, which showed that the autophagy related genes ATG2, *ATG3*, *ATG4A*, *ATG4B*, *ATG7*, *ATG9*, *ATG13*, *ULK1*, ULK2, *MAP1LC3A*, and *MAP1LC3B* were highly expressed in PGCs. Cells cultured in a medium differentiated with resveratrol stimulate the formation and accumulation of autolysosomes [18]. Therefore, we hypothesized that autophagy plays a regulatory role in the differentiation of ESCs into PGCs. In fact, our experiment supported this inference because PGCs formation was greatly inhibited after autophagy inhibition in vivo and in vitro. This was detected by CVH, which is one of the specific marker proteins for chicken reproduction. Further experiments showed that inhibition of autophagy greatly affected the differentiation of chicken ESCs. This is consistent with the results reported by Xu et al. [27]. Interfering with or knocking out LAMP2A in ESCs augments the expression of pluripotency factors (~80–140%) and AP reactivity (~60–80%). This change also delayed the differentiation induced by LIF withdrawal, slowed the downregulation of pluripotency factors and AP reactivity, and reduced the upregulation of differentiation markers. Our results fill a gap in the field of autophagy, which affects the differentiation of reproductive stem cells in poultry.

Rapamycin was used to activate autophagy during the differentiation of ESCs into PGCs but did not promote PGCs formation. It is well known that PGCs originate from ESCs and migrate to the gonad through a fixed pathway, which is a complex differentiation process including proliferation, differentiation, and migration; these processes may be carried out simultaneously and are regulated by factors such as mTOR signaling. Rapamycin activates autophagy by targeting mTOR. In addition to playing a central role in autophagy regulation, mTOR is also involved in many other important cellular functions, such as inhibition of protein translation, mitochondrial biogenesis, cell growth, motility, and proliferation. Studies have shown that rapamycin regulates cell differentiation and proliferation. Yu et al. [28] found that rapamycin reduces MG-63 cell viability and inhibits cell proliferation by activating autophagy. Yu et al. [28] found that rapamycin activated autophagy and inhibited HPC differentiation. Zhou et al. [29] found that the inhibition of mTOR in human ESCs by rapamycin resulted in a significant decrease in the expression of transcription factors (Pou5f1/Oct4 and SOX2), which promoted the development of the mesoderm and endoderm while inhibiting cell proliferation. Therefore, whether mTOR simultaneously regulates autophagy, cell differentiation, and proliferation in the complex process of chicken PGCs formation remains unclear and needs to be further explored.

## 5. Conclusions

Our results explored the relationship between autophagy and chicken PGCs for the first time, laying the foundation for studying the mechanism of autophagy in chicken PGCs and informing bird gene editing and the rescue of endangered birds.

## Figures and Tables

**Table 1:** Primer sequence of the genes.

Name	Primer sequence (5′-3′)
*Cvh*	F: GCAACTTCGGTAGCATCA
	R: TTGGACAGCCATTTCTTC
*C-kit*	F: GTCGCCGCTGGACTGATGT
	R: ATAAGGAAGTTGCGTTGG
*Oct4*	F: GGAGCAGTTTGCCAAGGACC
	R: ATAGAGCGTGCCCAGAGCC
*Nanog*	F: AGACCACCCATCTCACCG
	R: GCCTTCCTTGTCCCACTCT
*Lin28*	F: CCGAGAATGAGTCCCAACC
	R: TGAGTCCAGCATCGCACC
*β-Actin*	F: CAGCCATCTTTCTTGGGTAT
	R: CTGTGATCTCCTTCTGCATCC

## Data Availability

RNA-seq data that support the findings of this study have been deposited in the GEO database under accession code: GSE159511.
